# Effects of cariprazine on extracellular levels of glutamate, GABA, dopamine, noradrenaline and serotonin in the medial prefrontal cortex in the rat phencyclidine model of schizophrenia studied by microdialysis and simultaneous recordings of locomotor activity

**DOI:** 10.1007/s00213-018-4874-z

**Published:** 2018-04-11

**Authors:** Jan Kehr, Takashi Yoshitake, Fumio Ichinose, Shimako Yoshitake, Béla Kiss, István Gyertyán, Nika Adham

**Affiliations:** 1 Pronexus Analytical AB, Bromma, Sweden; 2 Department of Physiology and Pharmacology, Karolinska Institutet, Stockholm, Sweden; 3 Pharmacological and Safety Research, Gedeon Richter Plc, Budapest, Hungary; 4 Present Address: MTA-SE NAP B Cognitive Translational Behavioral Pharmacology Group, Budapest, Hungary; Department of Pharmacology and Pharmacotherapy, Semmelweis University, Budapest, Hungary; Institute of Cognitive Neuroscience and Psychology, Research Center for Natural Sciences, MTA, Budapest, Hungary; 5 Allergan, Madison, NJ USA

**Keywords:** Cariprazine, Aripiprazole, Schizophrenia, Rat phencyclidine model, Microdialysis

## Abstract

**Rationale:**

Aberrant glutamatergic, dopaminergic, and GABAergic neurotransmission has been implicated in schizophrenia. Cariprazine reverses the behavioral effects observed in the rat phencyclidine (PCP)-induced model of schizophrenia; however, little is known about its in vivo neurochemistry.

**Objectives:**

The study aims to compare the effects of cariprazine and aripiprazole on PCP-induced changes in the extracellular levels of glutamate, dopamine, serotonin, noradrenaline, and GABA in the rat medial prefrontal cortex (mPFC), and on locomotor activation.

**Methods:**

Microdialysis was performed in awake rats with probes placed into the mPFC. Rats (*n* = 7/group) received vehicle (saline), cariprazine (0.05, 0.2, or 0.8 mg/kg), or aripiprazole (3 or 20 mg/kg) via gavage. After 60 min, 5 mg/kg PCP was administered intraperitoneally (i.p.). Samples were taken before drug administration, during pretreatment, and after PCP injection. Locomotor activity recording and microdialysis sampling occurred simultaneously.

**Results:**

PCP treatment increased extracellular levels of all the neurotransmitters tested except GABA, for which there were no significant changes. Cariprazine and aripiprazole dose-dependently inhibited the PCP-induced increases of tested neurotransmitters. Overall effects were significant for higher cariprazine doses and both aripiprazole doses for glutamate and noradrenaline, for higher cariprazine doses and 20 mg/kg aripiprazole for dopamine, and for 0.8 mg/kg cariprazine and 20 mg/kg aripiprazole for serotonin and locomotor activity.

**Conclusion:**

Both cariprazine and aripiprazole dose-dependently attenuated PCP-induced hyperlocomotion and acute increases in glutamate, dopamine, noradrenaline, and serotonin levels in the mPFC; cariprazine was approximately 5-fold more potent than aripiprazole.

**Electronic supplementary material:**

The online version of this article (10.1007/s00213-018-4874-z) contains supplementary material, which is available to authorized users.

## Introduction

Schizophrenia is a debilitating, lifelong psychiatric disorder affecting approximately 1% of the population. Currently used antipsychotics are effective in improving positive symptoms but have relatively little benefit on the negative symptoms (e.g., social withdrawal and anhedonia) and cognitive deficits (Millan et al. 2016). Cariprazine (Vraylar™), a potent dopamine (DA) D_3_ and D_2_ receptor partial agonist with preferential binding to D_3_ receptors and partial agonism at serotonin 5-HT_1A_ receptors (Kiss et al. 2010), has recently been approved in the USA for the treatment of schizophrenia and bipolar mania in adults. It is also currently in clinical development for the treatment of bipolar depression and adjunctive treatment of major depressive disorder.

The D_3_ receptor is thought to play a role in mood and cognition, and it has recently emerged as a potential pharmacological target for neuropsychiatric disorders (Gross and Drescher 2012; Sokoloff et al. 2006). Cariprazine was developed based on the hypothesis that high affinity at D_3_ and D_2_ receptors may result in potent antipsychotic efficacy through D_2_ receptor blockade and confer additional D_3_ receptor-mediated benefits in the treatment of affective and cognitive deficits associated with schizophrenia and bipolar disorder (Gyertyán et al. 2008; Kiss et al. 2008). Cariprazine differs from currently used atypical antipsychotics (Ellenbroek and Cesura 2014) by having higher in vitro affinity and selectivity (almost an order of magnitude) for D_3_ versus D_2_ receptors (Kiss et al. 2010) and high levels of in vivo occupancy of both D_3_ and D_2_ receptors at antipsychotic-like effective doses in rats (Gyertyán et al. 2011) and clinically active dose ranges in patients with schizophrenia (Girgis et al. 2016). Other atypical antipsychotics, such as aripiprazole, clozapine, olanzapine, and risperidone, did not show significant D_3_ receptor occupancy at antipsychotic-like doses in rats (Kiss et al. 2012) or clinically relevant doses in patients (Caravaggio et al. 2014; Graff-Guerrero et al. 2009; Mizrahi et al. 2011). These data indicate that cariprazine can modulate in vivo D_3_ receptor activity to a greater extent than other antipsychotics.

Dysregulation in the glutamatergic, dopaminergic, and GABAergic neurotransmission systems may underlie the pathophysiological changes in the brain that lead to schizophrenia (Abi-Dargham et al. 2012; Kristiansen et al. 2006; Laruelle et al. 1996; Stan and Lewis 2012). NMDA receptor antagonists such as phencyclidine (PCP) induce psychopathology resembling the symptoms of schizophrenia in healthy individuals (Luby et al. 1959) and exacerbate schizophrenia symptoms in patients (Malhotra et al. 1997). PCP-based models have, therefore, routinely been used to model schizophrenia symptoms in animals (Javitt 1987; Neill et al. 2014; Sams-Dodd 1999). PCP is believed to produce adverse behavioral effects via blockade of the NMDA receptors on the GABAergic interneurons in the medial prefrontal cortex (mPFC) (Yonezawa et al. 1998), disinhibiting the cortico-cortical glutamatergic neurons (Berendse et al. 1992; Fonnum et al. 1981) and leading to increased glutamate (Glu) levels in the mPFC (Adams and Moghaddam 1998; Krystal et al. 2003; Moghaddam and Adams 1998). In addition to its effects on Glu efflux, PCP increases DA and serotonin (5-HT) release in the PFC (Hondo et al. 1994; Martin et al. 1998; Verma and Moghaddam 1996). Dysregulation of these monoamine neurotransmitter systems has also been implicated in schizophrenia (Carlsson 1978; Howes et al. 2015; Meltzer 1989).

We have previously demonstrated that in animal models of schizophrenia, cariprazine reversed PCP-induced behavioral effects (hyperlocomotion) (Gyertyán et al. 2011), demonstrating putative efficacy against positive symptoms of schizophrenia. In the follow-up study in mice, cariprazine significantly diminished the PCP-induced cognitive deficits in wild-type but not in D_3_ receptor knockout mice (Zimnisky et al. 2013). In addition, two recent studies provide further support for the ability of cariprazine to ameliorate cognitive and social deficits induced by PCP in adult rats (Neill et al. 2016) and in a PCP neurodevelopmental model of schizophrenia in rats (Watson et al. 2016). Together, the results from the PCP models of schizophrenia suggest that cariprazine may exert beneficial effects on the cognitive and social/affective functions disrupted by PCP, at least in part via its high affinity to the D_3_ receptors. Cariprazine displays subnanomolar affinity for the human D_3_ receptors, with approximately 5–10-fold selectivity over D_2L_, D_2S_, 5-HT_1A_, and 5-HT_2B_ receptor subtypes. It displayed much lower affinity for the adrenergic (α_1A_, α_1B_, α_1D_, α_2A_, β_1_, β_2_), 5-HT_2A_, histamine H_1_, 5-HT_7_, and 5-HT_2C_ receptors (Kiss et al. 2010). Using in vitro functional assays, cariprazine has shown an antagonist profile in G protein recruitment, and partial agonism in cAMP and β-arrestin signaling (Gao et al. 2015; Kiss et al. 2010). This unique receptor profile may account for the therapeutic efficacy of cariprazine. Indeed, cariprazine demonstrated enhanced efficacy for treating negative symptoms, compared with risperidone, in patients with predominant negative symptoms (Németh et al. 2017) and demonstrated efficacy versus placebo in patients with acute exacerbation of schizophrenia (Durgam et al. 2015; Durgam et al. 2014; Kane et al. 2015). Moreover, cariprazine has demonstrated efficacy in patients with bipolar depression and as adjunctive treatment in patients with major depressive disorder (Durgam et al. 2016a; Durgam et al. 2016b).

Considering the proven predictive validity of cariprazine in behavioral PCP models, it was of interest to examine to what extent cariprazine may modulate the PCP-induced increases in extracellular levels of the neurotransmitters Glu, DA, noradrenaline (NA), and 5-HT. There is little information on the in vivo neurochemistry of cariprazine. In the report of Kiss et al. (2010), cariprazine moderately increased DA turnover and slightly reduced 5-HT turnover in the mouse striatum, olfactory tubercles, and frontal cortex. Both cariprazine and aripiprazole, unlike risperidone, olanzapine, or haloperidol, produced greater enhancement of DA turnover and biosynthesis in the mouse limbic regions (olfactory tubercle) than in the striatum (Kiss et al. 2010). While these findings are indicative of the low propensity of cariprazine to induce extrapyramidal side effects, they do not predict the impact of cariprazine on neurotransmitter release and metabolism in the rodent model of schizophrenia.

The purposes of the present study were (1) to test the ability of cariprazine to modulate PCP-induced changes in the extracellular levels of neurotransmitters, including Glu, GABA, and the monoamines DA, NA, and 5-HT, as measured by microdialysis in the rat mPFC, while simultaneously recording their locomotor activity as a behavioral measure of antipsychotic-like effects and (2) to compare the effects of cariprazine on these endpoints to aripiprazole, a DA receptor partial agonist antipsychotic with higher affinity to D_2_ than to D_3_ receptors both in vivo and in vitro (Gyertyán et al. 2011; Kiss et al. 2010).

## Materials and methods

### Animals

Male Sprague Dawley rats (8–10 weeks of age, weighing 300–350 g at the day of experiment) were used in the study. The rats were received at approximately 200–250 g from Janvier Labs, France. Animals were allowed a minimum acclimatization period of 1 week prior to performing any experiments. No prophylactic or therapeutic treatment was administered during the acclimatization period. Animals were maintained in a controlled environment (22 ± 1 °C; 45–50% relative humidity) on a 12-h dark/12-h light (40 lx, lights on at 7:00 am) cycle. Room temperature and humidity were recorded continuously in the holding room. All rats were examined and weighed prior to initiation of the study to assure adequate health and suitability. Rats were randomly assigned to treatment groups.

All animal experiments and protocols were approved by the regional ethical committee at Stockholm County Court (Stockholms Norra djurförsöksetiska nämnd) following the directives of the Swedish Animal Welfare Act 1988:534 and complying with the Directive 2010/63/EU (Council of the European Parliament) “The Guide for the Care and Use of Laboratory Animals” and the “Principles of Laboratory Animal Care” (NIH Publications no. 85-23). All efforts were made to minimize animal suffering and the number of animals used for the study. The results are reported in accordance with the ARRIVE guidelines for reporting experiments involving animals (McGrath et al. 2010).

### Test compounds

Cariprazine hydrochloride salt and aripiprazole free base were provided by the Forest Research Institute, NJ, USA. PCP hydrochloride salt was purchased from LGC Standards (Boras, Sweden). All compounds were dissolved in saline on the day of the experiment.

### Groups and doses

Microdialysis experiments were carried out on six groups of seven rats each. Cariprazine and aripiprazole were administered orally (p.o.) to separate groups of rats; PCP was injected intraperitoneally (i.p.). Rats were treated with vehicle (saline, p.o.), cariprazine (0.05, 0.2, or 0.8 mg/kg, p.o.), or aripiprazole (3 or 20 mg/kg, p.o.). These doses of cariprazine have demonstrated an antipsychotic-like efficacy in a PCP-induced behavioral model (Gyertyán et al. 2011). As cariprazine was shown to be 5–20 times more potent than aripiprazole in behavioral tests, doses of 3 and 20 mg/kg were used in the present study. Sixty minutes after the p.o. administration, all groups received PCP (5 mg/kg, i.p.). All doses were calculated as a free base.

### Experimental procedures

#### Microdialysis

The microdialysis experiments were carried out on awake rats following the protocol described elsewhere (Kehr 1999; Kehr and Yoshitake 2006; Osborne et al. 1990).

#### Surgery and microdialysis experiments

Initially, the rats were anesthetized with isoflurane using a Univentor 400 anesthesia unit (AgnTho’s, Lidingö, Sweden) and placed in a stereotaxic frame (David Kopf Instruments, Tujunga, CA, USA) using a flat skull position with the incisor bar set to − 3.2 mm. During the operation, the body temperature of the animal was controlled by a thermometer and a heating pad maintained at 37 °C by the use of a CMA/150 temperature controller (CMA Microdialysis, Stockholm, Sweden). A middle scalp incision of 2–3 cm was made, and the flaps were held open using homeostatic forceps. After exposing the skull, a hole for the implantation of the guide cannula and three holes for the anchor screws were drilled using a fine trephine drill. Three microscrews were placed into the skull. A guide cannula (EICOM, Kyoto, Japan) was implanted into the mPFC at the following coordinates: AP + 3.2 mm, L + 0.5 mm, and V − 1.2 mm from the bregma and the brain surface, using the stereotaxic atlas of Paxinos and Watson (2007). The guide cannula was fixed firmly to the skull surface using dental cement (Dentalon Plus, Heraeus, Germany). The animals were allowed to recover for 5–7 days, kept individually in their home cages (Eurostandard type III H, Tecniplast, Italy) while maintaining visual, vocalization, and olfactory contact. During this period, the body weight and the general status of the animals were monitored on a regular basis.

On the day of the experiment, a microdialysis probe (EICOM A-I: 0.22 mm OD, 3 mm membrane length with 50 kDa cutoff) was inserted into the guide cannulae of the awake rat. The rat was placed into the system for freely moving animals (EICOM) equipped with a two-channel swivel (TCS2-23; ALS, Tokyo, Japan). The probes were perfused at a constant flow rate of 1 μl/min with artificial cerebrospinal fluid solution (148 mM NaCl, 4 mM KCl, 0.8 mM MgCl_2_, 1.4 mM CaCl_2_, 1.2 mM Na_2_HPO_4_, 0.3 mM NaH_2_PO_4_, pH 7.2). Following a 120-min stabilization period, the samples were collected every 30 min. Fifteen microliters of 0.1 M phosphate buffer (pH 3.0) containing 0.1 mM EDTA-2Na was pipetted into each 300-μl polypropylene sample vial placed in the refrigerated microfraction collector (EICOM). The first three 30-μl samples (collected from − 150 to − 60 min) were taken to measure the basal extracellular levels of Glu, GABA, DA, NA, and 5-HT. Thereafter, the drug or saline was administered p.o., and two samples were collected during an additional 60 min (− 60 to 0 min). At time 0 min, PCP (5 mg/kg, i.p.) was administered, and samples were collected for additional 3 h. After finalizing the experiment, the probe was removed. A microdialysis probe with its membrane removed acted as an infusion needle and was then inserted into the guide cannula. The infusion cannula was connected to a 10-μl Hamilton syringe with 2% aqueous solution of Evans blue dye, and 0.1 μl was infused into the brain by the use of a CMA/100 microinjection pump. The animals were sacrificed by an overdose of isoflurane and cervical dislocation. The brains were removed, fixed in 4% formalin in phosphate-buffered saline for 3–5 days, and cut in 60-μm sections for histological verification of the microdialysis probe placement.

#### Locomotor activity test

Locomotor activity was monitored by the use of a single-beam activity frame (44 × 30 cm ACTIMO 10, Shintechno, Japan) placed around the lower part of the Eurostandard type III cage. This arrangement allowed for simultaneous recordings of locomotor activity and microdialysis sampling. The data were collected by counting and summarizing the overall activity (number of beam crossings) in 5-min intervals and were further pooled in each respective 180-min sampling period, thereby corresponding to the microdialysis data expressed as the relative area under the curve (AUC_(0–180 min)_) values.

#### HPLC analysis

##### Glutamate and GABA

Amino acid neurotransmitters Glu and GABA were determined by precolumn derivatization with *ortho*-phthalaldehyde (OPA)/mercaptoethanol and isocratic elution reversed-phase column liquid chromatography with fluorescence detection following a minor modification of the protocol described elsewhere (Kehr 1998, 1999; Kehr and Yoshitake 2006). Briefly, the amino acid analyzer included a LC-10AD pump (Shimadzu, Kyoto, Japan), a LC-27A degasser (ALS, Tokyo, Japan), a CMA/200 refrigerated microsampler equipped with a 20-μl loop and operating at 6 °C, a L-7480 fluorescence detector (Hitachi, Tokyo, Japan), and the Clarity Data Acquisition System (DataApex, Prague, The Czech Republic). The analytical column was a 150 × 3.0 mm Eicompak SC-5ODS column (EICOM, Kyoto, Japan). The mobile phase was a mixture (34:66, *v*/*v*) of methanol and 0.1 M phosphate buffer (pH 6.0). The flow rate was 0.335 ml/min. The OPA/mercaptoethanol derivatization reagent was prepared daily from the OPA stock solution. Automated derivatization was carried out in the CMA/200 autosampler by dispensing and mixing the aliquot volumes of the sample and the reagent. Following the reaction time of 250 s, 10 μl was injected onto the column. The detection limit (signal-to-noise ratio = 3) for Glu and GABA was 10 fmol in the 10 μl injected onto the column.

##### DA, NA, and 5-HT

Monoamines 5-HT, NA, and DA were determined by ion-exchange narrow-bore column liquid chromatography with electrochemical detection as described elsewhere (Kehr 1999; Yoshitake et al. 2014). The chromatographic conditions were optimized to allow simultaneous determination of all three monoamines in the same sample. Briefly, a HPLC system, with an electrochemical detector (EICOM, Kyoto, Japan), and a CMA/200 refrigerated microsampler (CMA Microdialysis), equipped with a 20-μl loop and operating at 6 °C, were used. The electrochemical detector was equipped with a radial-flow electrochemical cell (EICOM) with the glassy carbon working electrode operating at the potential of + 450 mV versus the Ag/AgCl reference electrode. Monoamines were separated on a 200 × 2.0 mm ID column (CAX, EICOM). The mobile phase consisted of 0.1 M phosphate buffer at pH 6.0, 40 mM potassium chloride, 0.13 mM EDTA-2Na, and 30% (*v*/*v*) methanol. Under these conditions, the detection limits (signal-to-noise ratio = 3) for DA, NA, and 5-HT were 0.5, 0.6, and 0.5 fmol, respectively, in the 15 μl injected onto the column.

### Data presentation and analysis

Statistical analysis was performed using Prism 6 (GraphPad Software, USA) statistical software. The values are presented as means ± standard error of the mean (SEM), and differences are considered to be statistically significant at the *P* < 0.05 level. The basal extracellular levels were calculated and expressed as means ± SEM from seven rats in each group; the value for each rat in the respective group was calculated as the mean of three fractions collected from the − 120- to − 60-min period. For graphic representation of neurotransmitter outflow over time, the data were expressed as the percentage of the basal concentrations at time 0 min. The overall effects of the drug and vehicle treatments were expressed as the relative AUC value, defined here as the percentage of baseline values averaged over the 180-min post-treatment sampling period (rel. AUC_(0–180 min)_). Mean basal levels of the control and treatment groups were compared by using the Kruskal-Wallis test followed by Dunn’s multiple comparison test. Differences between the groups and treatments were analyzed by repeated measures two-way ANOVA followed by Bonferroni’s post test. Differences between the AUC_(0–180 min)_ values were compared by one-way ANOVA followed by Dunnett’s multiple comparison test.

## Results

### Probe placement in the mPFC

Histological verification of the microdialysis probe placement in rat brain sections revealed that the microdialysis probe membranes were placed exclusively in the mPFC, including cingulate, prelimbic, and infralimbic cortices.

### Basal extracellular levels of Glu, GABA, DA, NA, and 5-HT

The basal extracellular levels of Glu, GABA, DA, NA, and 5-HT in the rat mPFC in the saline-, cariprazine-, and aripiprazole-treated groups are summarized in Table 1. There were no significant differences between the mean basal levels of Glu, GABA, DA, NA, and 5-HT between the treated groups, with the exception of 5-HT, where the basal levels of the groups treated with cariprazine at 0.2 and 0.8 mg/kg were significantly lower (*P* < 0.05) than those of the control group. This could be partially explained by the variations in batches of the microdialysis probes, which could have lower recovery for these groups. In addition, the variations in estimated basal 5-HT levels could be caused by variations in chromatographic calibrations of 5-HT, which is the last eluting peak (16.2 min) in the chromatogram, and low (1–2 fmol/10 μl) concentrations of 5-HT are close to the limit of detection of the HPLC method. Other contributing factors, such as minor differences in animal experimental conditions, including the stabilization period after the probe insertion, between the tested groups, could not be excluded.

**Table 1:** Basal extracellular levels of Glu, GABA, DA, NA, and 5-HT in the rat mPFC in the saline-, cariprazine-, and aripiprazole-treated groups calculated from the first three samples (collected from − 150 to − 60 min) and expressed as mean ± SEM values, using the Kruskal-Wallis test followed by Dunn’s multiple comparison test

	Saline + saline	Cariprazine 0.2 mg/kg + saline	Saline + PCP	Cariprazine 0.05 mg/kg + PCP	Cariprazine 0.2 mg/kg + PCP	Cariprazine 0.8 mg/kg + PCP	Aripiprazole 3 mg/kg + PCP	Aripiprazole 20 mg/kg + PCP
Glu (pmol/10 μl)	7.21 ± 1.67	6.72 ± 1.41	5.01 ± 0.91	5.08 ± 0.62	10.06 ± 1.18	5.94 ± 0.99	5.40 ± 0.69	8.76 ± 2.42
GABA (fmol/10 μl)	227 ± 24.9	287 ± 42.1	257 ± 93.3	223 ± 50.0	193 ± 57.9	203 ± 44.7	205 ± 72.3	237 ± 51.32
DA (fmol/10 μl)	2.58 ± 0.278	2.93 ± 0.303	3.95 ± 0.386	3.72 ± 0.697	5.70 ± 1.423	2.53 ± 0.601	3.72 ± 0.969	3.71 ± 0.461
NA (fmol/10 μl)	8.13 ± 0.655	8.04 ± 0.754	8.31 ± 0.930	8.22 ± 1.02	8.79 ± 1.27	7.97 ± 0.993	8.00 ± 0.892	8.32 ± 0.347
5-HT (fmol/10 μl)	2.24 ± 0.227	2.93 ± 0.498	2.38 ± 0.249	1.38 ± 0.065	1.24 ± 0.118*	1.23 ± 0.094*	1.42 ± 0.167	1.44 ± 0.138

*5-HT* serotonin, *DA* dopamine, *GABA* gamma-aminobutyric acid, *Glu* glutamate, *NA* noradrenaline

*P < 0.05 compared to the saline + saline group

### Effects of cariprazine and aripiprazole on PCP-induced increase in extracellular levels of Glu and GABA

**Fig. 1:**
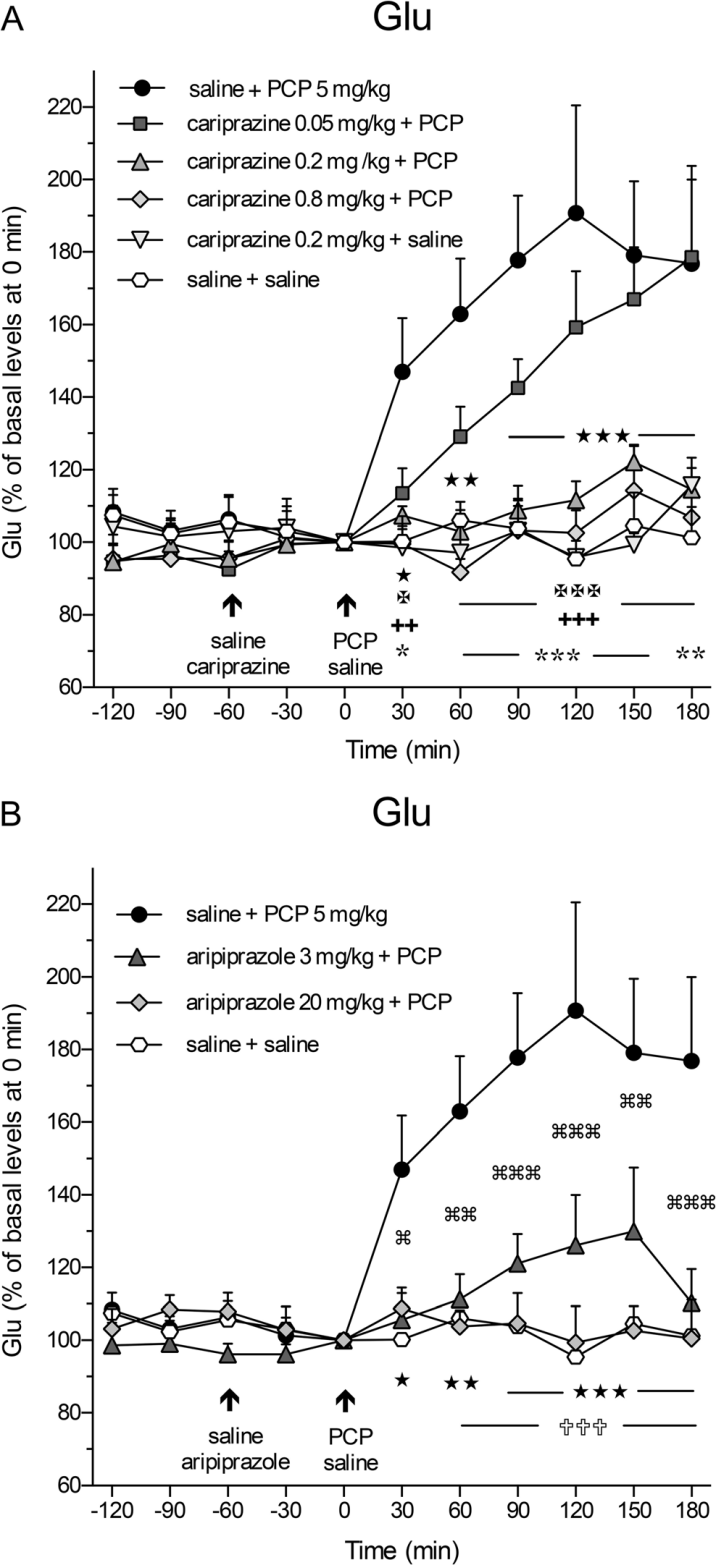
Effects of **a** cariprazine and **b** aripiprazole on the PCP-induced increase in extracellular levels of Glu in the mPFC of awake rats. **a** PCP significantly increased the Glu levels as compared to the saline + saline-treated group (hexagons; ★, *P* < 0.05; ★★, *P* < 0.01; ★★★, *P* < 0.001) and cariprazine 0.2 mg/kg + saline-treated group (triangles down; , *P* < 0.05; , *P* < 0.01; , *P* < 0.001). Cariprazine significantly attenuated the PCP-induced Glu efflux at 0.2 mg/kg (triangles up; ✠, *P* < 0.05; ✠✠✠, *P* < 0.001) and 0.8 mg/kg (diamonds; ✚✚, *P* < 0.01; ✚✚✚, *P* < 0.001), but not at 0.05 mg/kg (squares). **b** The significant PCP-induced increase in Glu levels (★, *P* < 0.05; ★★, *P* < 0.01; ★★★, *P* < 0.001) was significantly attenuated by aripiprazole at both 3 mg/kg (triangles; , *P* < 0.05; , *P* < 0.01; , *P* < 0.001) and 20 mg/kg (diamonds; ✞✞✞, *P* < 0.001) starting 60 min after the PCP injection

Administration of PCP (5 mg/kg, i.p.) at time 0 min caused a rapid and significant (*P* < 0.05) increase in extracellular Glu levels already in the first 30-min samples and reached the maximal value of 191 ± 30% at 120 min as compared to the control (saline + saline)-treated group (Fig. 1a). Pretreatment with cariprazine 60 min before PCP administration significantly attenuated the PCP-induced Glu release starting already at 30 min after the PCP injection when cariprazine was given at doses of 0.2 mg/kg (*P* < 0.05) and 0.8 mg/kg (*P* < 0.01), but not at 0.05 mg/kg. Notably, cariprazine (0.2 mg/kg + saline) administered alone had no effect on basal Glu levels. The results of the statistical analysis using two-way repeated measures ANOVA followed by Bonferroni’s post test are summarized in Table 2. The analysis (*F* and *P* values) revealed the significant effects of interaction time and treatment, treatment, and time. Similarly, pretreatment with aripiprazole at both tested doses significantly (*P* < 0.05 for 3 mg/kg and *P* < 0.01 for 20 mg/kg) attenuated the PCP-induced Glu release starting at 30 and 60 min after PCP injection, respectively, as shown in Fig. 1b and summarized in Table 2.

**Table 2:** Two-way repeated measures ANOVA followed by Bonferroni’s multiple comparison test for the values of Glu, GABA, DA, NA, and 5-HT and the locomotor activity

	Time × treatment		Treatment		Time	
Microdialysis data (time course, % of control)						
	*F*_(50,300)_	*P*	*F*_(5,30)_	*P*	*F*_(10,300)_	*P*
Cariprazine						
Glu	3.682	< 0.0001	8.175	< 0.0001	11.88	< 0.0001
GABA	1.142	n.s.	0.693	n.s.	0.453	n.s.
DA	6.226	< 0.0001	11.82	< 0.001	26.85	< 0.0001
NA	8.734	< 0.0001	13.25	< 0.0001	32.62	< 0.0001
5-HT	2.774	< 0.0001	4.557	< 0.01	10.06	< 0.0001
	*F*_(30,210)_	*P*	*F*_(3,21)_	*P*	*F*_(10,210)_	*P*
Aripiprazole						
Glu	4.045	0.0001	8.297	0.001	5.976	0.0001
GABA	1.183	n.s.	1.142	n.s.	0.725	n.s.
DA	7.049	0.0001	18.85	0.0001	23.62	0.0001
NA	7.835	0.0001	12.54	0.001	33.52	0.0001
5-HT	2.261	0.001	8.289	0.001	5.89	0.0001
Locomotor activity (time course, counts/5 min)						
	*F*_(325,1950)_	*P*	*F*_(5,30)_	*P*	*F*_(65,1950)_	*P*
Cariprazine	1.728	< 0.0001	5.643	< 0.001	4.608	< 0.0001
	*F*_(195,1365)_	*P*	*F*_(3,21)_	*P*	*F*_(65,1365)_	*P*
Aripiprazole	2.627	< 0.0001	4.683	< 0.02	5.027	< 0.0001

The groups were pretreated with cariprazine or aripiprazole, followed by PCP treatment, and compared to the saline + PCP-treated group. The microdialysis data were calculated as the relative values expressed as the percentage of the basal levels at time 0 min for each respective group; the locomotor activity was counted in 5-min bins (not significant (n.s.), *P* > 0.05)

*5-HT* serotonin, *DA* dopamine, *GABA* gamma-aminobutyric acid, *Glu* glutamate, *NA* noradrenaline

**Fig. 2:**
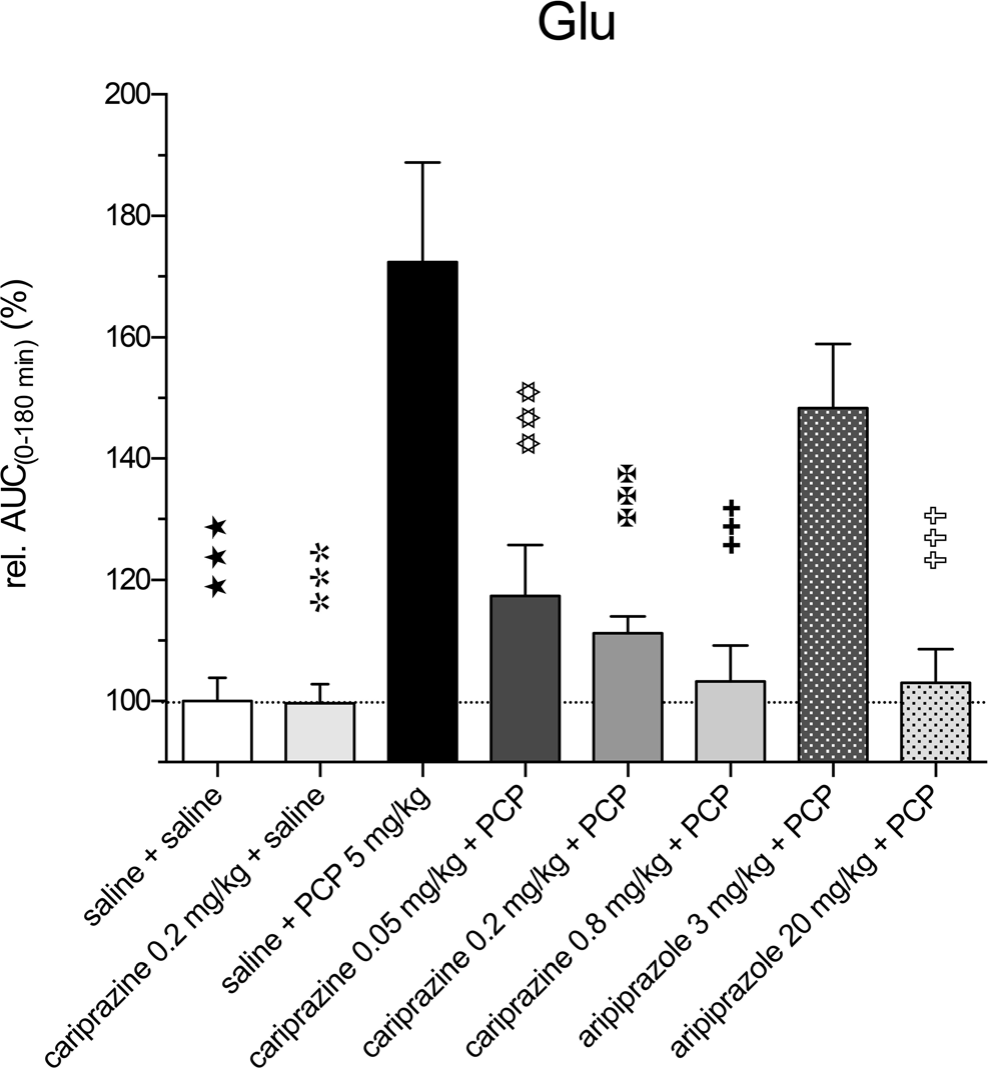
Overall effects of cariprazine and aripiprazole on the attenuation of the PCP-induced increase in Glu levels expressed as the relative AUC_(0–180 min)_ values of the drug-treated groups and compared to the saline + PCP group. Compared to the saline + PCP-treated group, relative AUC_(0–180 min)_ values were significantly lower for the saline + saline-treated group (★★★, *P* < 0.001), for the cariprazine 0.2 mg/kg + saline-treated group (, *P* < 0.001), for all three cariprazine doses (✡✡✡, ✠✠✠, ✚✚✚, *P* < 0.001), and for the aripiprazole-treated group at 20 mg/kg (✞✞✞, *P* < 0.001) but not at 3 mg/kg

The overall effects of cariprazine and aripiprazole on the attenuation of the PCP-induced increase in Glu levels (expressed as the relative AUC_(0–180 min)_ values of the drug-treated groups and compared to the saline + PCP group) are shown in Fig. 2a. One-way ANOVA followed by Dunnett’s multiple comparison test revealed that the relative AUC_(0–180 min)_ values were significantly lower (*P* < 0.001) for the saline + saline-treated group and cariprazine 0.2 mg/kg + saline-treated group when compared to the saline + PCP-treated group. Likewise, there were significant differences in the AUC_(0–180 min)_ values of Glu between the groups treated with 0.2 and 0.8 mg/kg cariprazine (*P* < 0.001 for both doses) and 20 mg/kg (*P* < 0.001) aripiprazole as compared to the saline + PCP-treated group. As both doses of cariprazine caused approximately the same effect, i.e., the Glu levels in these groups were not significantly different from the levels in the control group (Fig. 2a), it could be concluded that the intermediate (0.2 mg/kg) dose of cariprazine was sufficient to elicit the maximal effect on the attenuation of the PCP-induced increase in extracellular Glu levels in the rat mPFC.

Administration of PCP at time 0 min had no significant impact on the extracellular GABA levels. There was only a tendency towards decreased values; the lowest level, 79 ± 8% of the controls, was achieved at 120 min (Supplementary Figure 1A). Pretreatment with cariprazine or aripiprazole resulted in no significant differences between the treated groups (Table 2; Supplementary Figure 1B).

### Effects of cariprazine and aripiprazole on PCP-induced increases in extracellular levels of DA, NA, and 5-HT

**Fig. 3:**
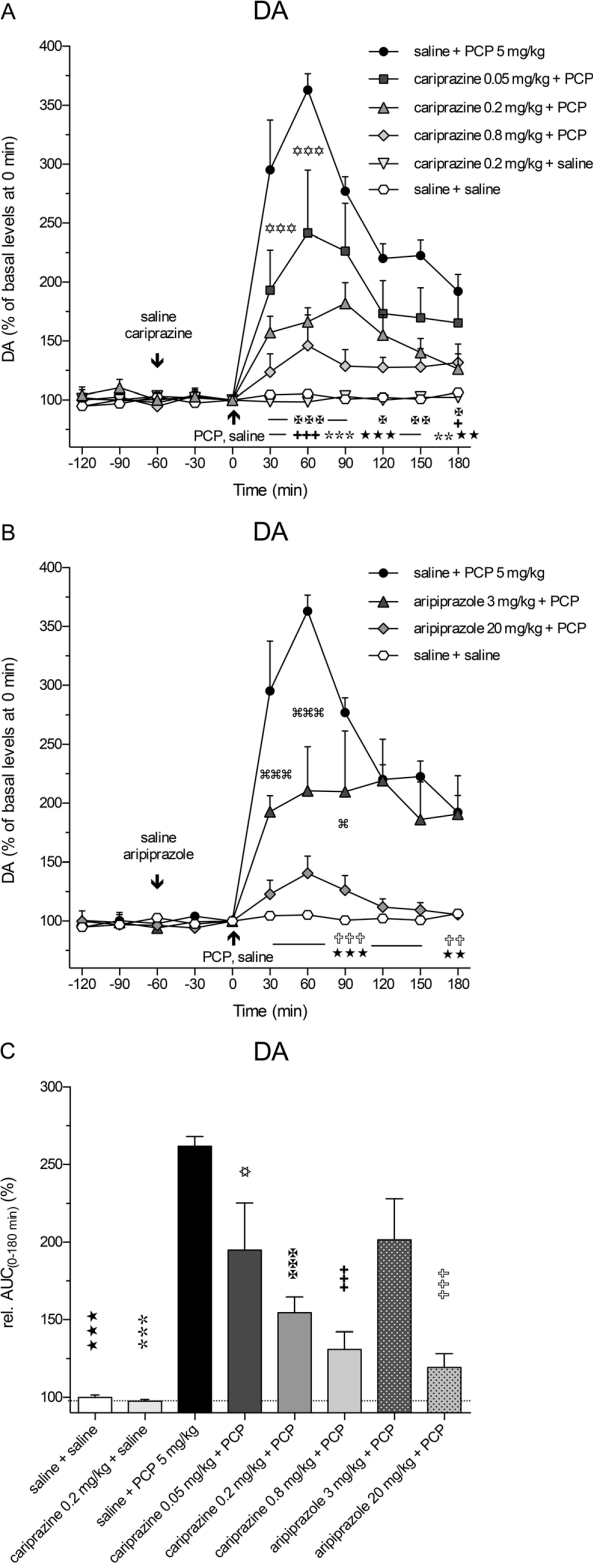
Effects of cariprazine and aripiprazole on the PCP-induced increase in extracellular levels of DA in the mPFC of awake rats. **a** PCP significantly increased the DA levels as compared to the saline + saline-treated group (hexagons; ★★, *P* < 0.01; ★★★, *P* < 0.001) and cariprazine 0.2 mg/kg + saline-treated group (triangles down; , *P* < 0.01; , *P* < 0.001). Cariprazine significantly attenuated PCP-induced DA release at 0.05 mg/kg (squares; ✡✡✡, *P* < 0.001), 0.2 mg/kg (triangles up; ✠, *P* < 0.05; ✠✠, *P* < 0.01; ✠✠✠, *P* < 0.001), and 0.8 mg/kg (diamonds; ✚, *P* < 0.05; ✚✚✚, *P* < 0.001). **b** Aripiprazole significantly attenuated PCP-induced DA release at 3 mg/kg (triangles; , *P* < 0.05; , *P* < 0.001) and 20 mg/kg (diamonds; ✞✞, *P* < 0.01; ✞✞✞, *P* < 0.001). **c** Compared to the saline + PCP-treated group, relative AUC_(0–180 min)_ values were significantly attenuated for the saline + saline-treated group (★★★, *P* < 0.001), for the cariprazine 0.2 mg/kg + saline-treated group (, *P* < 0.001), for all three cariprazine doses (✡, *P* < 0.05; ✠✠✠, ✚✚✚, *P* < 0.001), and for the aripiprazole-treated group at 20 mg/kg (✞✞✞, *P* < 0.001)

Administration of PCP at time 0 min caused a rapid increase in extracellular DA levels at 30 min (*P* < 0.001) and reached the maximal value of 363 ± 14% of the control DA levels at 60 min (Fig. 3a). Pretreatment with cariprazine at all three doses significantly (*P* < 0.001) attenuated the PCP-induced DA release starting at 30 min after the PCP injection. Similarly, pretreatment with aripiprazole significantly (*P* < 0.001, both for 3 and 20 mg/kg) attenuated the PCP-induced DA release starting at 30 min after the PCP injection (Fig. 3b). The results of statistical analysis with the corresponding *F* and *P* values are listed in Table 2. The DA levels in a separate group treated with cariprazine 0.2 mg/kg + saline were not different from the levels of the control group, confirming that cariprazine alone had no effect on the basal DA levels in the rat mPFC. The overall effects of cariprazine and aripiprazole on the attenuation of the PCP-induced increase in DA levels, expressed as the relative AUC_(0–180 min)_ values of the drug-treated groups and compared to the saline + PCP group, are shown in Fig. 3c. There were significant differences in the AUC_(0–180 min)_ values of DA between the groups treated with cariprazine at 0.05 mg/kg (*P* < 0.05), at 0.2 and 0.8 mg/kg (*P* < 0.001 for both), as well as for the group treated with 20 mg/kg aripiprazole (*P* < 0.001) as compared to the saline + PCP-treated group. The two higher doses of cariprazine caused similar effects.

**Fig. 4:**
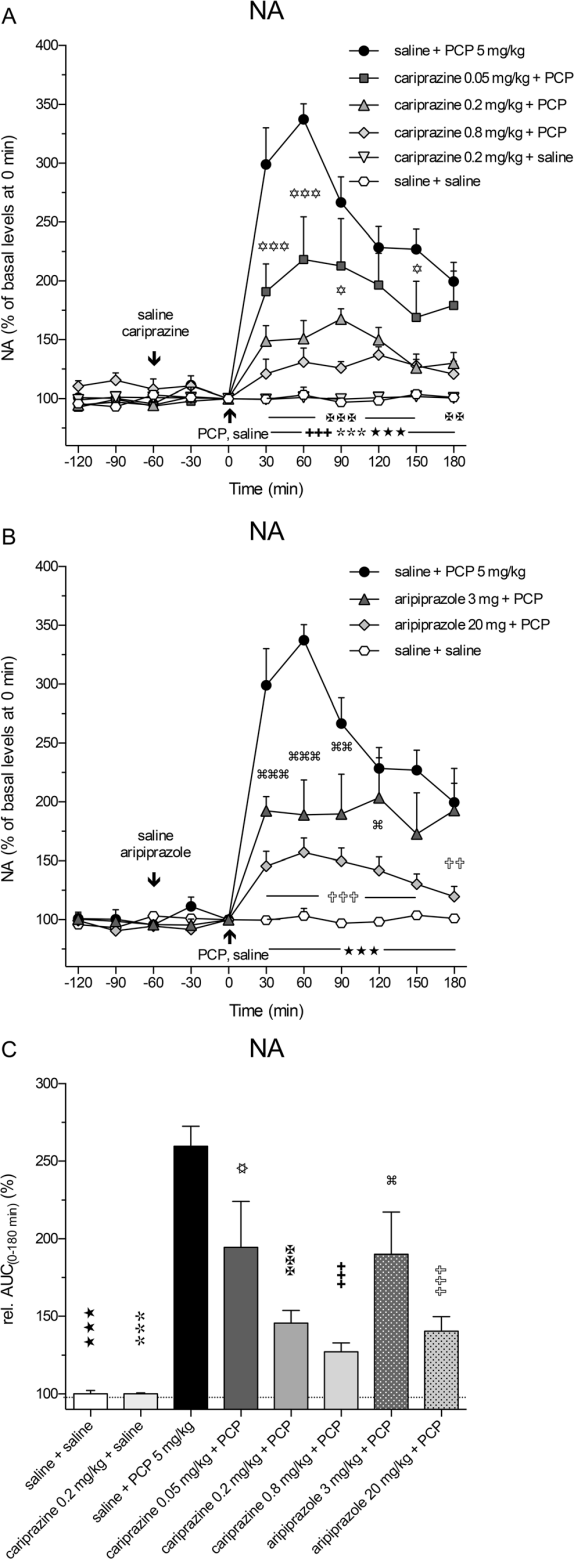
Effects of cariprazine and aripiprazole on the PCP-induced increase in extracellular levels of NA in the mPFC of awake rats. **a** PCP significantly increased the NA levels as compared to the saline + saline-treated group (hexagons; ★★★, *P* < 0.001) and cariprazine 0.2 mg/kg + saline-treated group (triangles down; , *P* < 0.001). Cariprazine significantly attenuated PCP-induced NA release at 0.05 mg/kg (squares; ✡, *P* < 0.05; ✡✡✡, *P* < 0.001), 0.2 mg/kg (triangles up; ✠✠, *P* < 0.01; ✠✠✠, *P* < 0.001), and 0.8 mg/kg (diamonds; ✚✚✚, *P* < 0.001). **b** Aripiprazole significantly attenuated PCP-induced NA release at 3 mg/kg (triangles; , *P* < 0.05; , *P* < 0.01; , *P* < 0.001) and 20 mg/kg (diamonds; ✞✞, *P* < 0.01; ✞✞✞, *P* < 0.001). **c** Compared to the saline + PCP-treated group, relative AUC_(0–180 min)_ values were significantly attenuated for the saline + saline-treated group (★★★, *P* < 0.001), for the cariprazine 0.2 mg/kg + saline-treated group (, *P* < 0.001), for all three cariprazine doses (✡, *P* < 0.05, ✠✠✠, ✚✚✚, *P* < 0.001), and for both aripiprazole-treated groups, 3 mg/kg (, *P* < 0.05) and 20 mg/kg (✞✞✞, *P* < 0.001)

PCP also markedly and significantly (*P* < 0.001) increased the extracellular levels of NA already in the first 30-min fraction and reached the maximal value of 337 ± 13% of the control levels at 60 min. Pretreatment with cariprazine significantly (*P* < 0.001) attenuated the PCP-induced NA release, starting with the first fraction collected at 30 min after the PCP injection (Fig. 4a). Cariprazine was effective at all three doses tested, whereas cariprazine (0.2 mg/kg) administered alone had no effects on the basal NA levels.

**Fig. 5:**
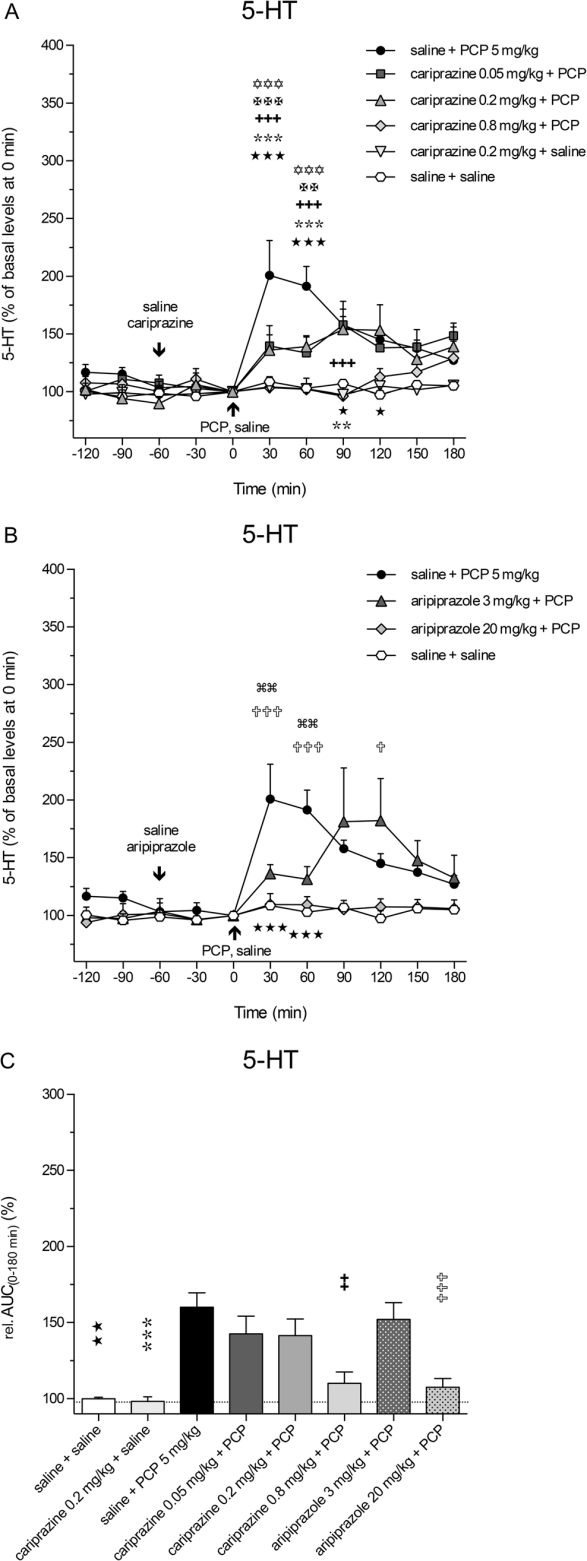
Effects of cariprazine and aripiprazole on the PCP-induced increase in extracellular levels of 5-HT in the mPFC of awake rats. **a** PCP significantly increased the 5-HT levels as compared to the saline + saline-treated group (hexagons; ★, *P* < 0.05; ★★★, *P* < 0.001) and cariprazine 0.2 mg/kg + saline-treated group (triangles down; , *P* < 0.01; , *P* < 0.001). Cariprazine significantly attenuated PCP-induced 5-HT release at 0.05 mg/kg (squares; ✡✡✡, *P* < 0.001), 0.2 mg/kg (triangles up; ✠✠, *P* < 0.01; ✠✠✠, *P* < 0.001), and 0.8 mg/kg (diamonds; ✚✚✚, *P* < 0.001). **b** Aripiprazole significantly attenuated PCP-induced DA release at 3 mg/kg (triangles; , *P* < 0.01) and 20 mg/kg (diamonds; ✞, *P* < 0.05; ✞✞✞, *P* < 0.001). **c** Compared to the saline + PCP-treated group, relative AUC_(0–180 min)_ values were significantly attenuated for the saline + saline-treated group (★★, *P* < 0.01), for the cariprazine 0.2 mg/kg + saline-treated group (, *P* < 0.001), and for the highest doses of cariprazine (✚✚, *P* < 0.01) and aripiprazole (✞✞✞, *P* < 0.001)

Likewise, pretreatment with aripiprazole at both tested doses significantly (*P* < 0.001) attenuated the PCP-induced NA release, starting at 30 min after the PCP injection (Fig. 4b). The statistical analysis results and the corresponding *F* and *P* values are listed in Table 2. The overall effects of cariprazine and aripiprazole on the attenuation of the PCP-induced increase in NA levels, expressed as the relative AUC_(0–180 min)_ values of the drug-treated groups and compared to the saline + PCP group, are shown in Fig. 5c. The AUC_(0–180 min)_ values were significantly lower for the groups treated with 0.05 mg/kg (*P* < 0.05), 0.2 mg/kg (*P* < 0.001), and 0.8 mg/kg (*P* < 0.001) cariprazine, similar to the groups treated with aripiprazole at 3 mg/kg (*P* < 0.05) and 20 mg/kg (*P* < 0.001) as compared to the saline + PCP-treated group. Both higher cariprazine doses caused similar effects, as already illustrated for Glu and DA.

The extracellular levels of 5-HT in the rat mPFC were also increased following the systemic administration of PCP, but to a lesser extent, than did those of DA and NA. The maximal increase, to 201 ± 30% (*P* < 0.001) of the control 5-HT levels, was achieved at 30 min (Fig. 5a). Pretreatment with cariprazine at all three doses significantly attenuated the PCP-induced 5-HT release in the 30-min (*P* < 0.001) and 60-min (*P* < 0.01 and *P* < 0.001) fractions, and the highest dose completely abolished the PCP effect on 5-HT release observed during the first 90 min after PCP injection. Cariprazine (0.2 mg/kg) administered alone had no effects on the basal 5-HT levels. Pretreatment with aripiprazole at both tested doses significantly (*P* < 0.01 for 3 mg/kg and *P* < 0.001 for 20 mg/kg) attenuated the PCP-induced 5-HT release starting at 30 min after the PCP injection (Fig. 5b). The statistical analysis results and the corresponding *F* and *P* values are listed in Table 2. The overall effects of cariprazine and aripiprazole on the attenuation of the PCP-induced increase in 5-HT levels, expressed as the relative AUC_(0–180 min)_ values of the drug-treated groups and compared to the saline + PCP group, are shown in Fig. 5c. The AUC_(0–180 min)_ values were significantly lower only for the highest doses of cariprazine (*P* < 0.01) and aripiprazole (*P* < 0.001).

### Effects of cariprazine and aripiprazole administered in combination with PCP on locomotor activity of rats

**Fig. 6:**
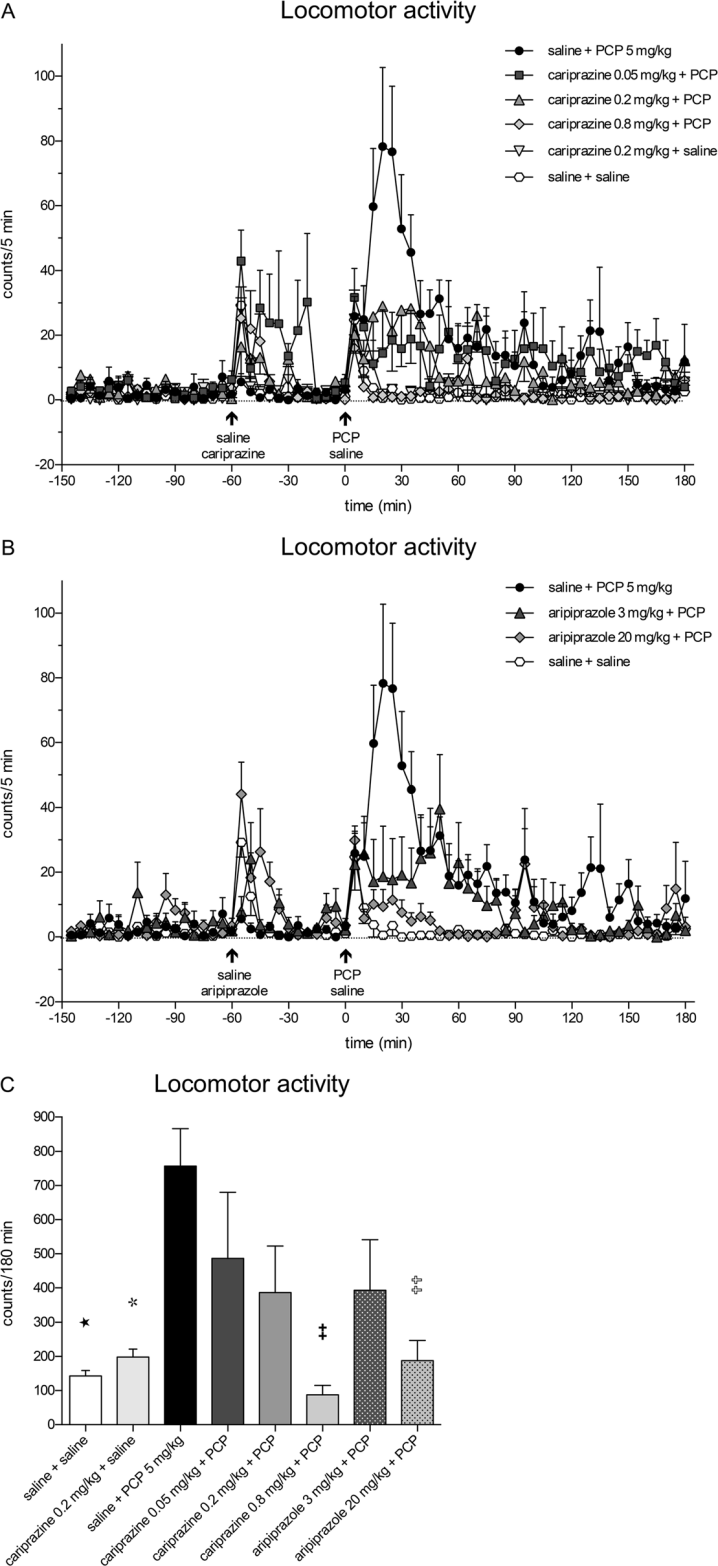
Effects of cariprazine and aripiprazole on PCP-induced increase of locomotor activity in rats undergoing microdialysis sampling. **a** Cariprazine significantly attenuated the peak of PCP-induced locomotor activation during the 15–35-min period for the 0.8 mg/kg dose (*P* < 0.001) and the 20–25-min period for the 0.05 and 0.2 mg/kg doses (*P* < 0.001). **b** Aripiprazole significantly attenuated the PCP-induced locomotor activation during the 15–25-min period for the 3 mg/kg dose (*P* < 0.001) and the 15–35-min period for the 20 mg/kg dose (*P* < 0.001). **c** PCP significantly increased the locomotor activity of rats as compared to the saline + saline-treated group (★, *P* < 0.05) and cariprazine 0.2 mg/kg + saline-treated group (, *P* < 0.05). Compared to the saline + PCP-treated group, cariprazine at 0.8 mg/kg (✚✚, *P* < 0.01) and aripiprazole at 20 mg/kg (✞✞, *P* < 0.01) significantly attenuated the total motor activity, expressed in counts/180 min

Administration of PCP caused a rapid increase in the locomotor activity of rats undergoing microdialysis sampling, reaching the maximal value of 78.3 ± 24.4 counts/5 min at 20 min (Fig. 6a). The PCP-induced increase was markedly higher and more prolonged than the increases in motor activity caused by the handling stress and the stress caused by the oral administration of saline or the test compounds at time − 60 min and saline at 0 min. Pretreatment with cariprazine significantly (*P* < 0.001) attenuated the PCP-induced motor activation during the period from 15 to 25 min post PCP injection for all three doses. The highest dose completely abolished (*P* < 0.001) the PCP-induced locomotion for an additional 10-min period (the 15–35-min period post PCP injection) (Fig. 6a). Similar effects were observed for aripiprazole administered at doses of 3 and 20 mg/kg (Fig. 6b). The statistical analysis results using two-way repeated measures ANOVA followed by Bonferroni’s post test and the corresponding *F* and *P* values are listed in Table 2. The overall effects of cariprazine and aripiprazole on the attenuation of the PCP-induced increase in locomotor activity during the entire sampling period of 180 min and compared to the corresponding value of the saline + PCP group are shown in Fig. 6c. The values were significantly lower only for the highest doses of cariprazine (*P* < 0.01) and aripiprazole (*P* < 0.01).

## Discussion

The results of the present microdialysis study show that acute oral treatment with cariprazine dose-dependently attenuated the PCP-induced increases in the extracellular levels of Glu, DA, NA, and 5-HT in the mPFC. Cariprazine alone (tested only as 0.2 mg/kg) had no effect on Glu, DA, NA, and 5-HT levels or motor activity. Cariprazine at oral doses of 0.05, 0.2, and 0.8 mg/kg attenuated the increase in locomotor activity induced by PCP in rats undergoing microdialysis sampling; this effect was only statistically significant at the 0.8 mg/kg dose. PCP caused only a modest, non-significant decrease in GABA levels; therefore, a clear tendency of cariprazine and aripiprazole to reverse this effect could not be statistically confirmed. Similar observations were made for aripiprazole, which was chosen as a reference substance and evaluated at doses of 3 and 20 mg/kg, p.o., in separate groups of rats.

In analogy to the use of PCP rodent models to evaluate antipsychotic drugs in behavioral tests, microdialysis can provide a mechanistic insight into the in vivo changes in neurotransmitter signaling and the role of the respective receptors and circuits involved.

The initial studies explored the role of the metabotropic Glu receptors (mGluRs) in modulating Glu release in the rat mPFC. The increase in Glu efflux and locomotor activation and stereotypy induced by PCP were abolished in rats that were pretreated with the mGluR II agonist LY354740, whereas the DA levels in the mPFC and nucleus accumbens remained increased (Moghaddam and Adams 1998). In the following study, the same authors concluded that clozapine, haloperidol, and the 5-HT_2A_ receptor antagonist M100907 did not effectively block the PCP-induced Glu release (Adams and Moghaddam 2001). M100907 was not found to be active during clinical trials in schizophrenia, suggesting a critical role for other neurotransmitters and their receptors in the pharmacological profile of current antipsychotic drugs (Ebdrup et al. 2011). NRA0045, a potent D_4_, 5-HT_2A_, and α_1_ adrenoceptor antagonist, inhibited the PCP-induced Glu release in the rat mPFC (Abekawa et al. 2003). Likewise, single administration of clozapine attenuated PCP-induced Glu release and hyperlocomotion at both 30-min (Abekawa et al. 2006) and 48-h (Abekawa et al. 2007) periods before PCP injection. In the following study, rats were chronically treated with PCP, and chronic clozapine treatment significantly attenuated both the PCP-induced increase in cortical Glu efflux and the reduction in GABA markers parvalbumin and GAD67 (Amitai et al. 2012).

In addition, local infusion of PCP decreased the GABA levels in the rat PFC (Yonezawa et al. 1998), and both systemic and local PCP potently inhibited the potassium-stimulated GABA release in the rat striatum (Hondo et al. 1995). In our study, we observed a modest, insignificant decrease in basal extracellular GABA concentrations following systemic PCP challenge.

The importance of the afferent inputs to the PFC and their role in the systemic effects of the NMDA receptor antagonists cannot be excluded. This is of particular significance when considering the NMDA receptor antagonist-induced disinhibition of the glutamatergic outputs from the mPFC neurons. Thus, local infusion of ketamine via a microdialysis probe in the mPFC did not increase Glu but did increase DA levels, whereas local infusion of NMDA increased Glu while decreasing DA release (Lorrain et al. 2003). Likewise, local perfusion of the AMPA receptor antagonist LY293558 through a probe placed in the ventral tegmental area (VTA) inhibited the PCP-induced locomotor activation and cortical, but not accumbal, DA release (Takahata and Moghaddam 2003).

The major finding in our study is that both cariprazine and aripiprazole dose-dependently attenuated the PCP-induced Glu efflux in the rat mPFC, similar to the prototype atypical antipsychotic drug clozapine (Abekawa et al. 2006; Amitai et al. 2012). The highest doses of cariprazine (0.8 mg/kg) and aripiprazole (20 mg/kg) almost completely abolished the PCP-induced effects on Glu and 5-HT levels and the locomotor activity of rats undergoing microdialysis sampling. However, oral aripiprazole was previously shown to have no effect on basal Glu levels in the rat mPFC (Carli et al. 2011). The DA and NA levels were also significantly and dose-dependently attenuated but remained increased between 136 and 157% of the basal levels even after the highest doses of cariprazine and aripiprazole. Previous studies have shown that aripiprazole at a low dose (0.3 mg/kg) increased DA levels, and higher doses had no effect in either rats (Li et al. 2004) or mice (Zocchi et al. 2005). The effect of cariprazine and aripiprazole on PCP-induced 5-HT efflux is in agreement with the study of Amargos-Bosch et al. (2003) showing that clozapine and olanzapine, but not haloperidol, suppressed the 5-HT efflux elicited by PCP or ketamine in the mPFC of rats. This study, together with the NRA0045 antagonist data (Abekawa et al. 2003), supports the hypothesis that the blockade of the 5-HT_2A_ receptors and α_1_ adrenoceptors by atypical antipsychotic drugs may contribute to the blockade of the PCP-induced increase in cortical 5-HT and Glu efflux. PCP-induced stimulation of the AMPA receptors could be attenuated by the local perfusion of the AMPA receptor antagonist LY293558 through probes in both the PFC and VTA (Takahata and Moghaddam 2003). Stimulation of the mPFC with local infusion of S-AMPA was reversed by 5-HT_2A_ receptor antagonists (Amargos-Bosch et al. 2003), providing a functional link between the NMDA, AMPA, and 5-HT_2A_ receptors, i.e., between the efflux of Glu and 5-HT elicited by PCP.

Cariprazine and aripiprazole have similar binding affinities to the rat 5-HT_2A_ and 5-HT_1A_ receptors, whereas cariprazine is about 10 times less potent than aripiprazole at the human 5-HT_2A_ receptors (Kiss et al. 2010). However, both cariprazine and aripiprazole are more potent antagonists at the 5-HT_2A_ receptors than clozapine, which may account for their robust effect on PCP-induced Glu and 5-HT release, and locomotor activity. The effects of cariprazine and aripiprazole on PCP-induced locomotor activity may not be due to a non-specific attenuation of spontaneous activity producing catalepsy and/or sedation as our previous findings showed no effect of these compounds on these parameters (Gyertyán et al. 2011). Clozapine was shown to attenuate PCP-induced hyperlocomotion, but only partially (Abekawa et al. 2006). There was a shift in the time course profiles of DA release in the mPFC and in the locomotor activity of rats following systemic PCP administration (Adams and Moghaddam 1998). Our results are in line with this observation, showing the markedly delayed and sustained increases in Glu, DA, NA, and 5-HT levels compared to the locomotor activity. PCP induced the maximal increase in forward locomotion between 20 and 25 min post injection, whereas the peak effects for the monoamines occurred at 60 min for DA and NA and 30 min for 5-HT. One possible limitation of the study is the difference in the temporal resolution of the behavioral recordings, counting the activity in 5-min bins, and the microdialysis sampling in 30-min intervals. Regardless of the differences in time intervals for monitoring the behavioral and neurochemical endpoints, these findings indicate that PCP-induced locomotor activation precedes the elevation of Glu levels in the mPFC. The prolonged increase in DA efflux is not capable of sustaining the locomotion, as reported elsewhere (Adams and Moghaddam 1998).

In conclusion, both cariprazine and aripiprazole dose-relatedly attenuated PCP-induced hyperlocomotion and acute increases in Glu, DA, NA, and 5-HT levels in the mPFC, with cariprazine displaying a much greater potency than aripiprazole. The potency of cariprazine was approximately fivefold higher than that of aripiprazole when comparing the doses of 0.2 mg/kg cariprazine and 3 mg/kg aripiprazole corrected for their bioavailability. The bioavailability of cariprazine and aripiprazole in rat is 53% (Gyertyán et al. 2011) and 16% (EMA/737723/2013), respectively, which correspond to effective doses of 0.106 and 0.48 mg/kg, respectively. As acute PCP (or ketamine) has been proven to model the psychotic, cognitive, and negative symptoms of schizophrenia, cariprazine may have benefits for improving cognitive deficits and negative symptoms of schizophrenia in addition to being an effective antipsychotic agent. Interestingly, recent clinical results demonstrated the increased efficacy of cariprazine over risperidone in patients with predominant negative symptoms (Németh et al. 2017). Future studies are needed to explore the role of cariprazine’s unique receptor profile, including D_3_ receptor activity, in the treatment of negative symptoms and cognitive deficits of schizophrenia.

## Electronic supplementary material

ESM 1 (PDF 181 kb)
