# Supplementary material for: Effects of cariprazine on extracellular levels of glutamate, GABA, dopamine, noradrenaline and serotonin in the medial prefrontal cortex in the rat phencyclidine model of schizophrenia studied by microdialysis and simultaneous recordings of locomotor activity
Source: Psychopharmacology (Berl). 2018 Apr 11;235(5):1593–607. doi: 10.1007/s00213-018-4874-z (PMC5920013; doi:10.1007/s00213-018-4874-z)
Supplement: Supplementary file 1 — (PDF 181 kb) [file 213_2018_4874_MOESM1_ESM.pdf]

Electronic Supplementary Material

Title: Effects of cariprazine on extracellular levels of glutamate, GABA, dopamine, noradrenaline and serotonin in the medial prefrontal cortex in the rat phencyclidine model of schizophrenia studied by microdialysis and simultaneous recordings of locomotor activity

Journal: Psychopharmacology

Authors: Jan Kehr[1,2], Takashi Yoshitake[2], Fumio Ichinose[1], Shimako Yoshitake[1,2], Béla Kiss[3], István Gyertyán[3,*], Nika Adham[4]

[1]Pronexus Analytical AB, Bromma, Sweden

[2]Department of Physiology and Pharmacology, Karolinska Institutet, Stockholm, Sweden

[3]Pharmacological and Safety Research, Gedeon Richter Plc, Budapest, Hungary

[4]Allergan, Madison, NJ 07940, USA

*Present Affiliation: MTA-SE NAP B Cognitive Translational Behavioral Pharmacology Group, Budapest, Hungary; Department of Pharmacology and Pharmacotherapy, Semmelweis University, Budapest, Hungary; Institute of Cognitive Neuroscience and Psychology, Research Center for Natural Sciences, MTA, Budapest, Hungary

Corresponding author: Jan Kehr, PhD, Professor adjunct; e-mail: jk@pronexus.se

Supplementary Fig. 1 Effects of cariprazine and aripiprazole on the extracellular levels of GABA in the mPFC of awake rats. A) Administration of PCP (5 mg/kg, i.p.) at time 0 min had no significant impact on the extracellular GABA levels. There was only a tendency to decrease the values; the lowest level of 79 ± 8% of the controls was achieved at 120 min. B) PCP slightly but not significantly decreased the basal GABA levels; pretreatment with cariprazine and aripiprazole showed a tendency to reverse this effect, which was significant for cariprazine at the highest dose (✚ , $P < 0.05$).

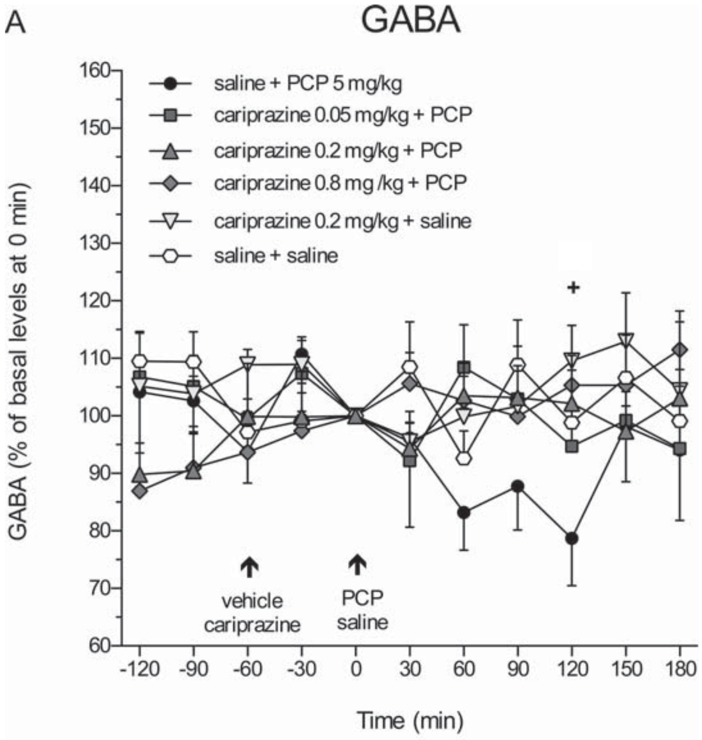
A
GABA
saline + PCP 5 mg/kg
cariprazine 0.05 mg/kg + PCP
cariprazine 0.2 mg/kg + PCP
cariprazine 0.8 mg /kg + PCP
cariprazine 0.2 mg/kg + saline
saline + saline
GABA (% of basal levels at 0 min)
vehicle
cariprazine
PCP
saline
Time (min)
+

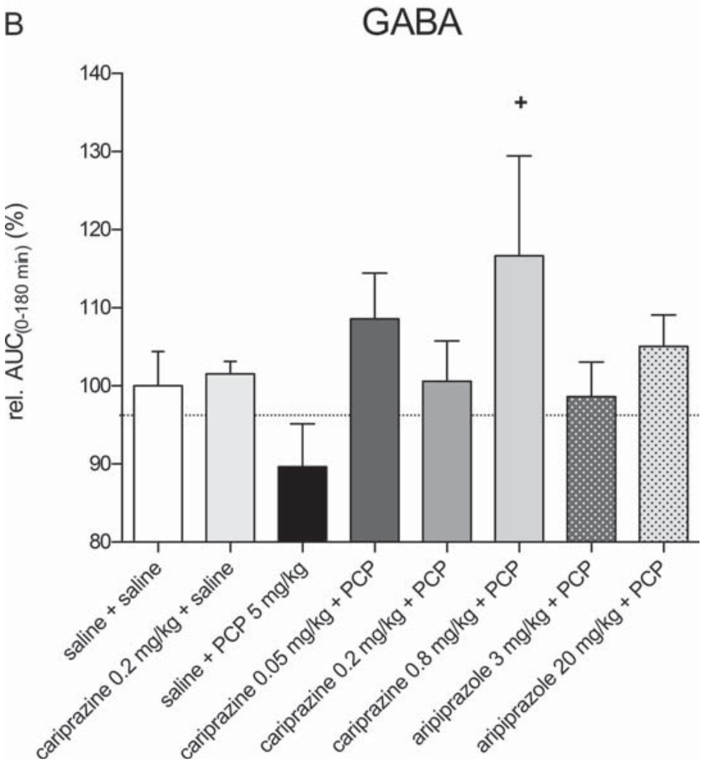
B
GABA
rel. AUC(0-180 min) (%)
+
saline + saline
cariprazine 0.2 mg/kg + saline
saline + PCP 5 mg/kg
cariprazine 0.05 mg/kg + PCP
cariprazine 0.2 mg/kg + PCP
cariprazine 0.8 mg/kg + PCP
aripiprazole 3 mg/kg + PCP
aripiprazole 20 mg/kg + PCP
